# Integrated Giant Magnetoresistance Technology for Approachable Weak Biomagnetic Signal Detections

**DOI:** 10.3390/s18010148

**Published:** 2018-01-07

**Authors:** Hui-Min Shen, Liang Hu, Xin Fu

**Affiliations:** 1 School of Mechanical Engineering, University of Shanghai for Science and Technology, Shanghai 200093, China; hmshen@usst.edu.cn; 2 State Key Laboratory of Fluid Power and Mechatronic Systems, Zhejiang University, Hangzhou 310028, China; xfu@zju.edu.cn

**Keywords:** giant magnetoresistance, biomagnetic signal, integration, 1/*f* noise, CMOS

## Abstract

With the extensive applications of biomagnetic signals derived from active biological tissue in both clinical diagnoses and human-computer-interaction, there is an increasing need for approachable weak biomagnetic sensing technology. The inherent merits of giant magnetoresistance (GMR) and its high integration with multiple technologies makes it possible to detect weak biomagnetic signals with micron-sized, non-cooled and low-cost sensors, considering that the magnetic field intensity attenuates rapidly with distance. This paper focuses on the state-of-art in integrated GMR technology for approachable biomagnetic sensing from the perspective of discipline fusion between them. The progress in integrated GMR to overcome the challenges in weak biomagnetic signal detection towards high resolution portable applications is addressed. The various strategies for 1/*f* noise reduction and sensitivity enhancement in integrated GMR technology for sub-pT biomagnetic signal recording are discussed. In this paper, we review the developments of integrated GMR technology for in vivo/vitro biomagnetic source imaging and demonstrate how integrated GMR can be utilized for biomagnetic field detection. Since the field sensitivity of integrated GMR technology is being pushed to fT/Hz^0.5^ with the focused efforts, it is believed that the potential of integrated GMR technology will make it preferred choice in weak biomagnetic signal detection in the future.

## 1. Introduction

Non-invasive detection of biomagnetic signals derived from active biological tissue, including magnetocardiograms (MCGs) [1,2,3], magnetoencephalograms (MEGs) [4,5,6], magnetospinograms (MSGs) [7,8], and magnetomyograms (MMGs) [9,10], has been a promising study method for biological phenomena with respect to high fidelity, temporal and spatial resolution. However, the weak biomagnetic signals can be easily polluted by environmental noise. Biomagnetic signals are normally sampled by strict detection systems in magnetically shielded rooms (MSRs). Since the biomagnetic fields attenuate rapidly by the distance to the neural sources, miniaturized sensors placed at closer range will provide stronger signals. With the extensive applications of biomagnetic source imaging (BMSI), approachable biomagnetic signal detection systems for point-of-use and point-of-care applications are attractive for both medical diagnostics and academic research [3,6,10]. During the past decades, there has been an increasing need of extensive biomagnetic signal applications in micro-dimensional detection for higher spatial resolution and system integration for real-time and robust processes. Traditional BMSI systems face challenges in further progress and enhancement. As a rapidly expanding field of medical research, the biomagnetism community has stressed that the development of alternative, miniaturized, and approachable biomagnetic detectors would constitute an important step towards the wider distribution of biomagnetism [11,12], e.g., clinical diagnoses, human-computer interaction (HCI), and education.

Giant magnetoresistive (GMR) sensors can realize reliable size-independent magnetic signal detection in the sub-nT range at room temperature using micron-sized structures. The large-scale fabrication process of GMR provides high capacity of integration with low power consumption and cost. These make GMR sensors good candidates for enhanced BMSI [13,14,15]. The GMR effect is a basic phenomenon that occurs in magnetic materials ranging from nanoparticles over multilayered thin films to permanent magnets. The discoverers of this phenomenon, Grünberg [16] and Fert [17], were awarded with the Nobel Prize in Physics in 2007. During the early stage, applications of GMR sensors were focused on industry [18,19,20] for information storage, which is now a well-established technology. High quality integrated GMR systems combined with multiple technologies have been proposed [21,22,23]. In recent years, an extensive research activity has been triggered to exploit the potentials of integrated GMR in ultra-low biomagnetic signal detection [21,22,23,24,25,26,27,28,29]. Without involving increased cost and complicated structure, integrated GMR brings aggregative performance improvements [23,28,29] in the fabrication process, structure size, anti-noise ability and sensitivity, taking advantages of multiple technologies and the inherent properties of GMR.

In this contribution, we intend to provide a review of integrated GMR for biomagnetic signal detection and demonstrate the feasibility of integrated GMR for approachable biomagnetic field applications. Firstly, heuristic models for GMR and biomagnetic signals are provided, and challenges in BMSI are highlighted. Then, we focus on the current developments in 1/*f* noise reduction and sensitivity enhancement of the integrated GMR technologies for comparable intensity to biomagnetic signals (sub-pT), to provide guidance for future development of the integrated GMR technology in BMSI. Finally, frontier research of integrated GMR systems in in vivo/vitro BMSI applications is presented, illustrating the application feasibility. It is expected that this review will be a valuable reference for GMR research in weak biomagnetic signal detections.

## 2. Integrated GMR Sensors towards BMSI

### 2.1. Physical Principles of GMR Multilayers

The GMR effect is a quantum mechanical magnetoresistance effect. The physical origin is related to spin-dependent scattering in magnetic multilayers, which introduced scientists to a new discipline termed spintronics [30]. Nowadays the underlying physics of GMR and the inter-layer exchange coupling are broadly understood [31,32]. GMR sensors with greater resistance variation are now available, among them the spin valve (SV) sensor is an advanced type of GMR sensor. A good review of the SV mechanism is given in [33].

**Figure 1:**
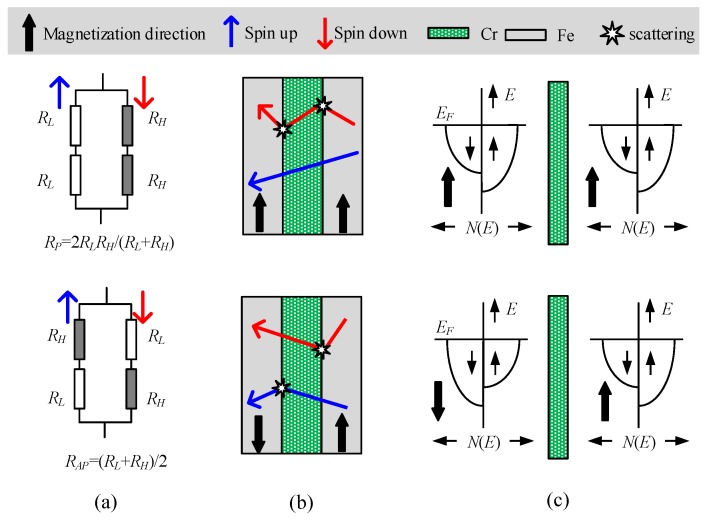
(**a**) Schematic of the resistor model; (**b**) Schematic of spin-dependent scattering at the interfaces between ferromagnetic and non-ferromagnetic layers with parallel magnetization (PM) and anti-parallel magnetization (APM); (**c**) Schematic of density of electronic states with PM and APM. *E*, the electron energy; *E_F_*, the Fermi level; *N*(*E*), density of states.

Based on the two current models proposed by Mott [34], the GMR effect can be understood by the simple resistor model (Figure 1a). In a multilayer structure including a pair of ferromagnetic thin film layers separated by a non-magnetic conducting layer (Figure 1b), the resistance change arises when the externally applied magnetic field aligns the magnetic moments of the successive magnetic layers. The conduction electrons in ferromagnetic layers transport via channels of spin-up and spin-down electrons. The spin-up and spin-down electrons with the spin direction parallel and antiparallel to the magnetization direction are termed ‘majority’ and ‘minority’ electrons, respectively. Based on the assumption that the electron mean free path for both spin channels are longer than the layer thickness, electrons with spin parallel to the layer magnetization are scattered in a different way to electrons with spin in the opposite direction to the layer magnetization. The spin-dependent scattering can be further explained with the density of state function for transition [35,36] (Figure 1c). As illustrated in Figure 1, stronger scattering of electrons with spin antiparallel to the magnetization direction produces a large resistance (represented by *R_H_*), while weaker scattering produces a small resistance (represented by *R_L_*) for the other spin direction. Consequently, the magnetoresistance ratio (MR) can be modeled by the simple resistor model expressed as:

(1) $$\frac{\Delta R}{R}=\frac{R_{AP}-R_{P}}{R_{P}}=\frac{{\left(R_{L}-R_{H}\right)}^{2}}{4R_{L}R_{H}},$$

### 2.2. Progress in GMR with High Integration

The simplicity of the GMR mechanism mentioned above is a great adventure for ultra-low biomagnetic signal detection. It has been proved that GMR sensors alone can realize pT (10^−12^T) (DC) magnetic field sensing at room temperature [37]. By the accurate control of the thin-film materials, interfaces and electrical characteristics, presently manufacture of GMR sensors with micro/nano dimensions is a mature technology with a solid footprint in a wide range of applications [13,15,18,38]. GMR based technology is the preferred choice for low magnetic fields detection with high spatial resolution. The maturity of GMR sensor fabrication relies on large-scale methods, and offers many advantages:

- Smart system integration with multiple components of Si-based integrated circuits in small platforms with decreased operation difficulty and power consumption, such as lab-on-a-chip devices [39,40], signal post-process modules and communication modules;
- Miniaturized structures without sensitivity loss providing increased spatial resolution in weak biomagnetic fields sensing;
- Room-temperature operation without bulky cooling systems and the associated expensive costs;
- Array applications with robust spatial measurements and compact systems [2,41];
- Real-time and multi-mode process based on high compatibility with standard CMOS processes, such as simultaneous electronic and magnetic readout [28,42].

**Figure 2:**
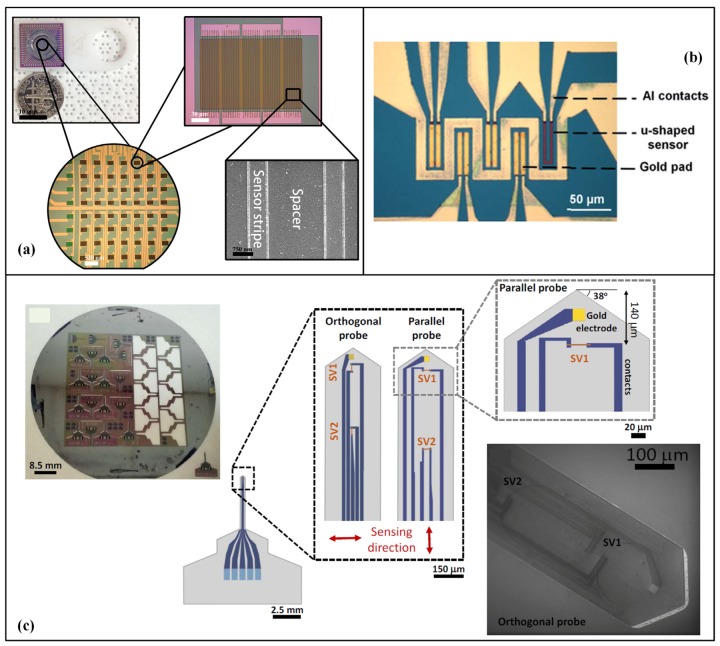
(**a**) Fabricated GMR chip for virus detection [39]; (**b**) GMR chips bonded on a printed circuit board for microfluidic platform (Adapted from [40] with permission of The Royal Society of Chemistry); (**c**) Integration of GMR sensors in needle shape micromachined silicon probes for neuronal magnetic field recording [28].

Typical applications of integrated GMR systems are illustrated in Figure 2. In Figure 2a [39], high sensitive detection of influenza virus using 8 × 8 GMR biosensor array (each GMR sensor has a size of 120 μm × 120 μm) was realized in real time without operator intervention. As presented in Figure 2b, an integrated system based on GMR sensor array was reported in [40] for the detection of cell-free DNA fragments (ALU115 and ALU247), taking advantages of integration and high sensitivity. In Figure 2c, a needle-shaped micromachined silicon probe with integrated GMR sensing elements and a thin film electrode was fabricated and successfully recorded simultaneous magnetic and electric neural signals. As an emerging field, applications of GMR sensors for BMSI have benefited from the merits of high sensitivity and high integration with multi-technologies.

## 3. BMSI towards High Resolution Portable Applications

### 3.1. Electrophysiological Basis of Biomagnetic Signals

**Figure 3:**
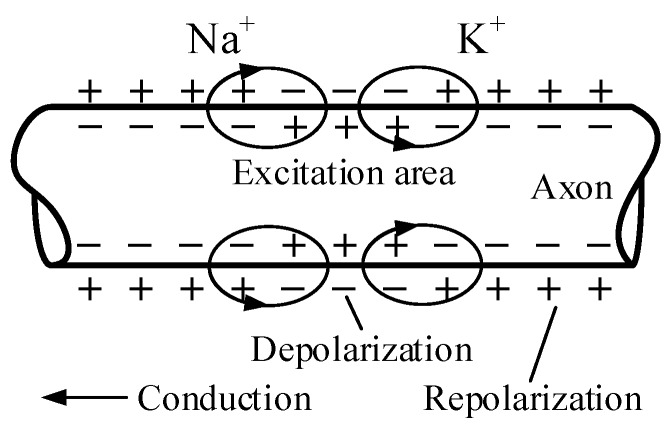
Transmission of action potential.

The physiological origin of biomagnetic signals is the electrochemical activity of body cells [43,44,45]. As illustrated in Figure 3, when nerve cells (related to MEG and MSG) or muscle cells (related to MCG and MMG) are excited, their cell membrane can produce electrochemical impulses and conduct them along the membrane. The ionic channels constitute an important part of the cell membrane. The flow of these ions in neurons or muscular fibers forms the basis of bioelectric phenomena, and exhibit as biomagnetic signals at macro level.

**Figure 4:**
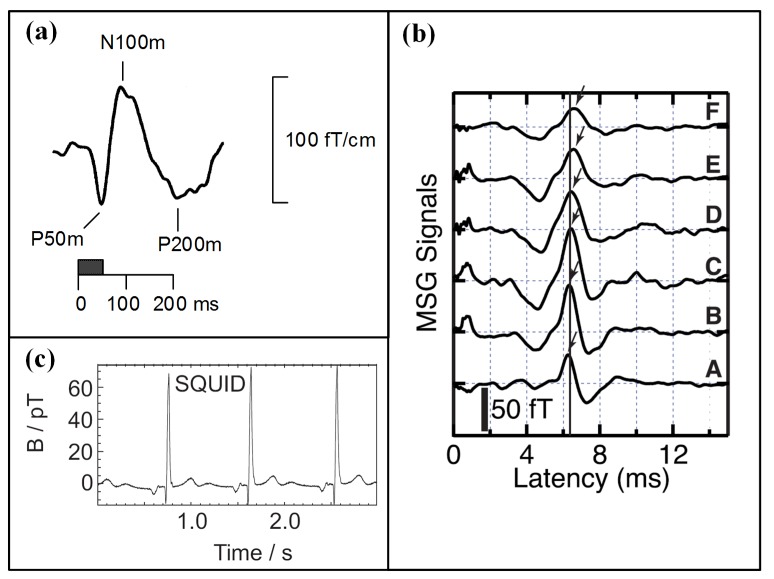
(**a**) Typical MEG response evoked by a 50-ms tone [44]; (**b**) Waveforms of the *z*-component of the MSG signals [46]; (**c**) Raw MCG signal measured by a SQUID at the chest center [47].

Typical biomagnetic signals derived from the electrochemical activity of nerve cells or muscle cells, termed MEG, MSG, or MCG, are presented in Figure 4. Since the magnetic permeability of biological tissue is nearly homogeneous and almost identical to the vacuum, no distortion is introduced to the biomagnetic signals of the body surface by internal anatomical structures. Besides, the magnetic measurement is not only non-invasive but also contact-less. Consequently, BMSI has been a promising method for biological phenomena detection of biological tissue and provides results with high temporal and spatial resolution.

### 3.2. Challenges for Approachable BMSI

As a promising and effective medical diagnostic method, nowadays BMSI has achieved wide recognition and can be found in most regular hospitals, although there are still challenges in further progress and promotion of BMSI for extensive applications.

(1). Extremely low magnitude order and signal-to-noise ratio

The quality of detected biomagnetic signals directly decides the performance of BMSI. However, the intensity of the biomagnetic signals are extremely low (about 100 fT (10^−15^T) for MEG [44] and MSG [8], and several tens of pT for MCG [48] and MMG [9]) and several orders smaller than the external interface. The measurements can be easily disturbed, for instance, the signal-to-noise ratio (SNR) in MEG equaling 1 is common [49], in spite of the expensive and sophisticated instruments. It should highlight that the sensor interference (1/*f* noise) largely degrades response linearity and low-frequency detection ability in GMR sensors [50], which is more difficult to suppress [51,52,53]. Various technologies have been studies to boost the SNR, including electromagnetic shielding techniques, reference channels, and signal processing [8,54,55,56].

(2). Low-cost, flexible and miniaturized detectors

The wide diffusion of BMSI relies greatly on development of low-cost, flexible and miniaturized detectors. During the past decades, measurements of the extremely low biomagnetic signal are largely dominated by superconducting quantum interference device (SQUID) with detection limit of 3fT/Hz^0.5^ at 4 K [6,52,53,57]. However, due to the hash cryogenic temperatures operating requirements, the SQUID is bulky and costly (a several million dollars device costing over 100,000 dollars in running costs per year). In addition, flexible and miniaturized sensor arrangement shows great potential to improve temporal and spatial resolutions, since the signal magnitude will be greater with the reduced distance between sensor and tissues [58,59]. Besides, microscopic recording of a small region is needed in study the asynchronous biomagnetic signals, where multiple time scales are involved during the electrochemical process of biomagnetic signal generation [60].

## 4. Integrated GMR Technologies with Enhanced Performance

Benefitting from the maturity of GMR technology in the performance (large-scale fabrication, reliability, micron size, room working temperature and low costs) and high integration with multiple technologies, integrated GMR systems for BMSI began to draw more attention in the past few decades. Although a GMR sensor in principle can reach really high sensitivity, taking advantage of size-independent resolution, the applications of integrated GMR systems are restricted, considering the harsh condition of signal measurements and challenges mentioned above in BMSI. New approaches for integrated GMR performance improvement focuses on noise reduction and sensitivity enhancement in order to realize comparable intensity detection to biomagnetic signals (sub-pT).

### 4.1. Elimination of 1/f Magnetic Noise in Low-Frequency Magnetic Sensing

**Figure 5:**
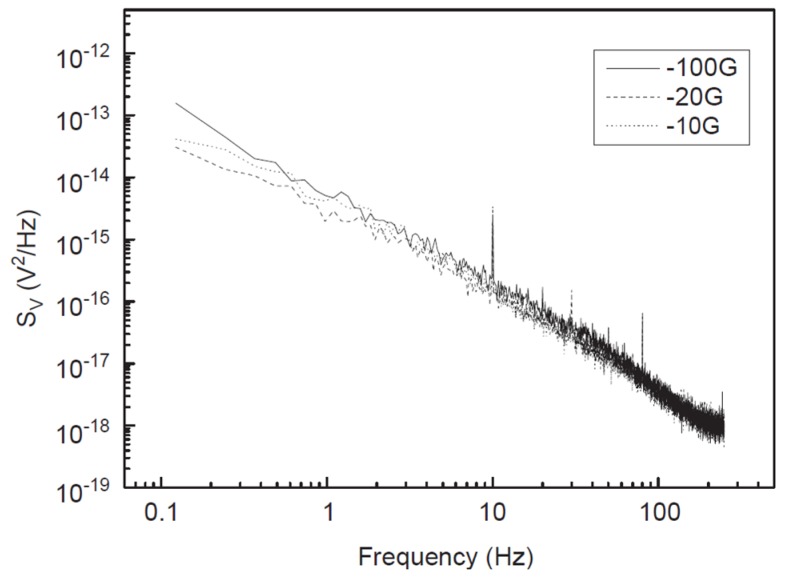
1/*f* noise in a yoke-type GMR with sensitivity of 0.4 nT/Hz^0.5^ for different external applied fields [63].

In principle, GMR sensors could compete with SQUID if the field gain or magneto-resistance can be increased largely [61], but in the low-frequency biomagnetic fields sensing, the detectable minimum field is greatly limited by the 1/*f* magnetic noise [62]. Figure 5 [63] shows recorded 1/*f* noise in a yoke-type GMR sensor at room temperature with different external applied field, illustrating that (a) the 1/*f* noise increases rapidly with the decrease of frequency and (b) there is no extra magnetic noise at low frequency. This noise originates from the fluctuations of energy around equilibrium due to the presence of magnetic domains [64,65], and it can be dependent on the shape, size and material properties of the device. Therefore, the maximum density of 1/*f* magnetic noise occurs in the linear transition of the sensor, where the magnetization of the sensing layer is switching between the two saturation states. The power spectral density *S_V_*(*f*) of 1/*f* noise in GMR sensor is proportion to 1/*f* and usually given by the Hooge relation [66,67]:

(2) $$S_{V}\left(f\right)=\gamma_{H}V^{2}/N_{C}f,$$

where *V* is the applied voltage and *N_C_* is the number of charge carrier in the active volume. The parameter *γ_H_* is called Hooge constant and it is used as comparison reference for 1/*f* noise.

The performance of GMR sensors in low-frequency biomagnetic field detection is severely limited by 1/*f* noise. To extend the lower detectable limit, many efforts have been contributed to eliminating the 1/*f* noise. Two kinds of 1/*f* noise elimination methods are reviewed below, including integrated GMR/microelectromechanical systems (MEMS) and optimized structure design. 

Approach I: Integrated GMR/MEMS Systems

In recent years, the method by integrating GMR sensors with MEMS magnetic flux modulation (MFM) shows great potential in 1/*f* noise reduction [50,68,69,70,71,72,73,74,75]. The introduction of MEMS MFM can mechanically modulate the quasi-static signal into a frequency above the 1/*f* knee, overcoming the higher noise spectrum density present at the low-frequency region [68,69,70,71]. Two kinds of MEMS MFM have been approached, including electrostatic MEMS and piezoelectric MEMS.

**Figure 6:**
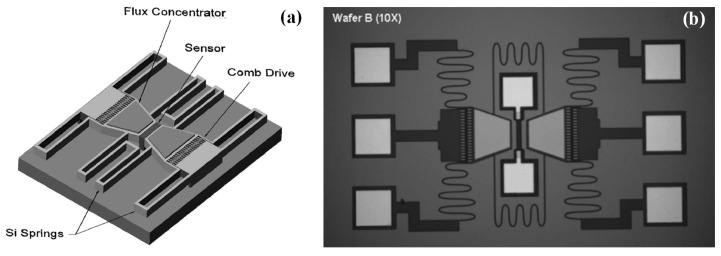
(**a**) Illustration of the concept of the MEMS MFM; (**b**) Scanning electron microscopy (SEM) picture of the MEMS MFM [71].

In the work presented by Edeltein [69,70,71], the employed electrostatic MEMS structure can realize one to three orders of 1/*f* noise magnitude reduction. As presented in Figure 6a, this system was fabricated on silicon-on-insulator wafers, and consisted of a pair of MFMs deposited on MEMS flaps driven by electrostatic comb drives and a SV sensor placed between them. The motion of the MFMs modulates the field at the position of the sensor and thus shifts the operating frequency. The prototype sensor was validated by shifting magnetic signals of 1.3 μT at 25 Hz to the high frequency range (around 48 kHz), and the results showed that the SNR was increased by a factor of 2 using an estimate of the noise at 1 Hz [71].

**Figure 7:**
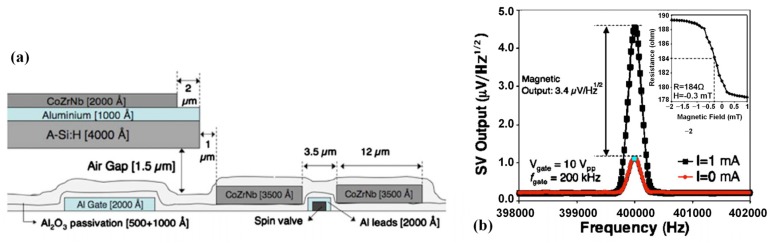
(**a**) Cross sectional view of the integrated sensor with all relevant features. (**b**) SV voltage output induced by the microcantilever at 200 kHz [68].

Similar research was implemented by Guedes et al. [68]. As shown in Figure 7a, a modulated AC field at the SV sensor was created by cantilever with flux guide oscillation with the operating frequency shifted from DC to high frequencies. The feasibility of this integrated system was validated by applying an AC signal of 10 V_p.p_ at 200 kHz to the cantilever gate electrode causing the microcantilever to oscillate at 400 kHz. The results in Figure 7b show that the magnetic output of the SV sensor is 3.4 μV/Hz^0.5^ with modulation efficiency *e*_cant_ = 0.11%, and a minimum 540 nT/ Hz^0.5^ detectable static magnetic field is reported.

Compared to electrostatic MEMS hybrid systems, hybrid system integrates GMR and piezoelectric MEMS can provide more advantages. Since no air-gap capacitor is required compared with capacitive MEMS, piezoelectric MEMS devices are more area-efficient with simpler geometry. Besides, lower driving voltage is required [76], and piezoelectric materials are reversible helping for cost reduction. All of these advantages make piezoelectric MEMS more attractive.

**Figure 8:**
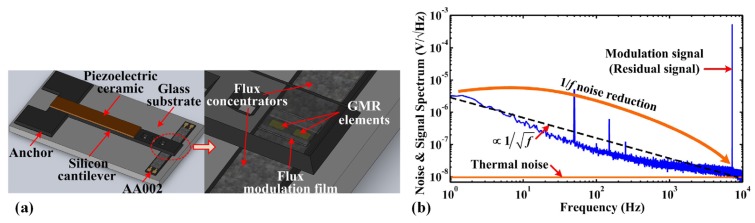
(**a**) Solid model of the vertical motion flux modulation structure [73]; (**b**) Noise and signal spectrum of the vertical motion flux modulation sensor without external magnetic field [75].

A vertical motion flux modulation scheme was developed by Hu et al. [50,73,74,75]. As illustrated in Figure 8a, the proposed structure is composed of a silicon cantilever with a piezoelectric ceramic and a soft magnetic film, which is simpler compared with the aforementioned structure. The magnetic field measured by GMR elements in the air gap is partly transferred to a higher frequency domain as a result of the flux modulation film vibration. Thus, high-frequency magnetic field with much lower 1/*f* noise can be detected, providing improved SNR. The power spectrum presented in Figure 8b shows that the noise level of the modulated magnetic field can be reduced from 2000 nV/Hz^0.5^ at 1 Hz to 10 nV/Hz^0.5^ around 7 kHz. The low-frequency detection ability of the prototype sensor can be improved to 120 pT/Hz^0.5^ at 1 Hz, which is about 83 times higher than the detection ability of used GMR elements (10 nT/Hz^0.5^ at 1 Hz) [50]. At a frequency of 7 kHz, the modulation efficiency of this structure can achieve 18.8%.

**Figure 9:**
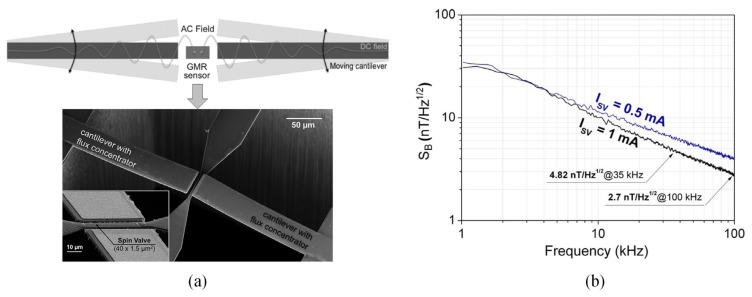
(**a**) The schematic and SEM picture of the integrated device; (**b**) Noise spectrum in magnetic units in the 1–100 kHz range for different biasing currents in the proposed sensor [72].

A parallel approach that combines a SV sensor, MFM, and A1N-based MEMS piezoelectric cantilevers was proposed in [72]. The schematic and SEM picture of the proposed device are presented in Figure 9a. If the measured low-frequency field at frequency *f*_1_ flows through the MFM on the MEMS cantilevers with a motion frequency *f*_0_, an oscillatory (AC) magnetic field at frequencies 2*f*_0_ ± *f*_1_ will be induced and detected by the SV sensor. MFM made by multilayers of antiferromagnetic coupled CoFeB layers ([CoFeB 38 Å/Ru 18 Å] × 32/CoFeB 38 Å) is placed on the surface of MEMS cantilevers, and modulate the low-frequency magnetic signals above 20 kHz. Based on the noise spectrum curve in Figure 9b we can get that the sensitivity of this integrated device was 301 nT/Hz^0.5^ for static field detection, and 602 nT/Hz^0.5^ for low-frequency fields if the loss from the amplitude modulated sidebands equals one half. The modulation efficiency with a 1 mT applied static field is about 1.59%, which can achieve 50% through finite element model simulations.

Approach II: Optimized Structure Design

**Figure 10:**
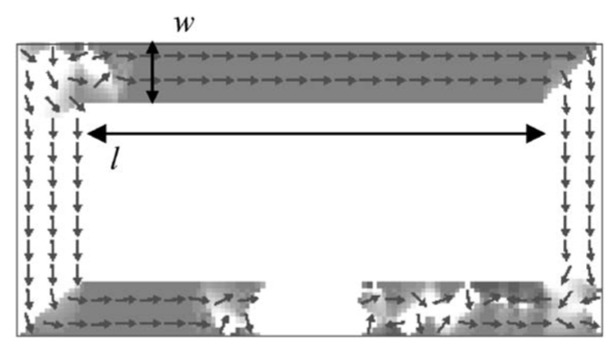
The magnetic configuration of the yoke after saturation and decrease to zero external field as obtained from micro-magnetic simulation [63].

The presence of magnetic domains in the active region is the dominant source of 1/*f* noise in low frequency for GMR sensors [63,64]. As illustrated in Figure 10, a yoke-type shape structure was reported in [63], where no magnetic domains exist in the active region. The configuration of the proposed spin valve sensor includes a soft layer made of NiFe(3.5)/CoFe bilayer (thicknesses are given in nm) and a hard layer made of CoFe/IrMn or CoFe/PtMn. According to the general power density formula presented in (2), the 1/*f* noise depends mainly on the parameter *Nc* in small structures and then is inversely proportional to the volume (1/*wl*)^0.5^. Besides, it has been verified that to keep the yoke stable with domains formation and a hysteretic behavior, *w* should be smaller than 12 μm. In case of good contacts on the GMR stacks, a power of 5 mW with a sensing current of 3 mA was used and the maximal resistance was chosen to provide high sensitivity. With the 1/*f* noise as the limitation, *l* should be then maximized.

Based on these remarks, a yoke-type sensor with *l* = 60 μm and *w* = 6 μm has been achieved, and the sensitivity of the proposed yoke-type GMR sensor at room temperature is 0.4 nT/Hz^0.5^. The 1/*f* noise in this yoke-type GMR sensor presented in Figure 5 shows that there is no difference between the 1/*f* noise in the saturated state (100 G) and in the sensitive area (10 G and 20 G). The noise measurements in two regimes in Figure 5 demonstrate that there is no extra magnetic noise at low frequency due to magnetic domain formation in the proposed yoke-type GMR sensor.

### 4.2. Sensitivity Enhancement for High Spatial Resolution

The unique power of BMSI is the localization of active neural areas with reasonable spatial resolution and excellent temporal accuracy. The biomagnetic fields are very weak and require sub-pT (MCG) or even fT (MEG) sensitivity level. Despite the 1/*f* noise, accurate measurements of raw ultra-low biomagnetic signals depend on high field sensitivity. Magnetic flux concentrator (MFC) can concentrate the external field in the sensor region with an appropriate geometry. The employment of MFCs decreases the linear operating range of GMR sensor without bringing additional noise [77]. The effective gain *G* introduced by the MFCs is defined by the ratio between the magnetic field in the gap and external field, which can reach hundreds [78]. The gain depends on the geometrical parameters, including length, yoke/pole ratio, and intrinsic magnetic properties of the material, including magnetic permeability. There are two types of MFC, including soft-ferromagnetic material-based MFC and superconducting-based MFC.

Approach I: Integration of GMR and Soft-Ferromagnetic Material-based MFC Systems

**Figure 11:**
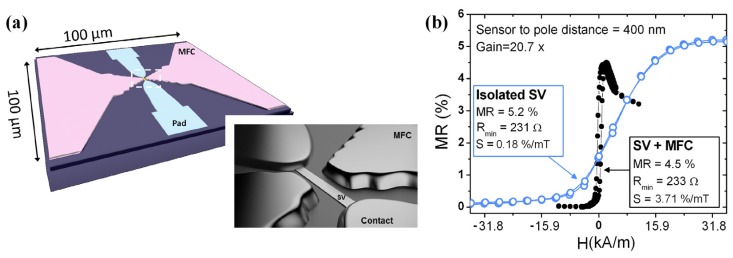
(**a**) Illustration of an integrated device combing SV sensor and MFCs; (**b**) Transfer curves comparing the isolated SV sensor with the corresponding device including the MFC [81].

MFC made of soft ferromagnetic materials (e.g., NiFe or amorphous Co based alloys) can realize significant sensitivity enhancement to pT level at room temperature [77,78,79,80,81]. In the work presented by Leitao et al. [81] (Figure 11), an ultra-compact sensor consisting of nanometric SV sensor placed within the gap of 6000 Å–thick thin-film MFC element was designed. As shown in Figure 11b, a maximum gain of 20.7 was obtained for pole-sensor distance of 400 nm with sensitivity increased from 0.18%/mT to 3.7%/mT. With the proposed microfabrication process, the patterned MFCs exhibited a clean liftoff profile (down to 380 nm), leading to an extremely small active sensing area.

Approach II: Integration of GMR and Superconducting Technology

**Figure 12:**
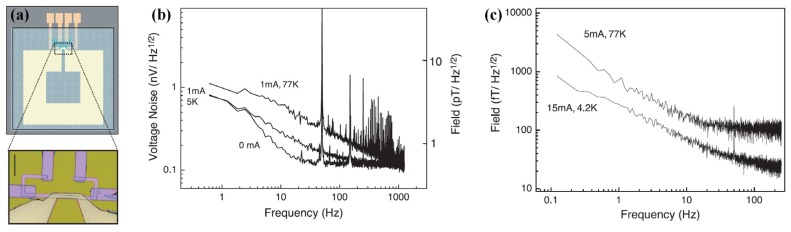
(**a**) Schematic view of the integrated device, comprising a GMR sensor with a superconducting loop; (**b**) The noise spectrum of the voltage output of the GMR and the field sensitivities; (**c**) The noise spectrum of the YBaCuO mixed sensor at 4.2 K and 77 K [25].

Superconducting MFC provides an extra field gain by transforming the weak field on a large surface to a strong field on a small surface based on the Meissner effect [25,26], and makes it possible to detect fT signals at low frequency (as illustrated in Figure 12a). In this kind of integrated detector, a supercurrent is induced by a magnetic signal and generates locally enhanced flux lines in a constriction, which can be detected by the GMR sensor placed above or below the constriction. The gain of this kind of integrated sensor device can reach over 1000 [27]. In the YBCO-based devices, the GMR can work with a higher current at 4.2 K. Figure 12b gives the noise spectral density for the niobium-based device with a sensing current of respectively 1 and 15 mA under noise level of 540 fT/Hz^0.5^. Figure 12c shows that the noise level of the YBCO-based device with the same sensing current is about 32 fT/Hz^0.5^. Comparison of Figure 12b,c demonstrates that the best sensitivity achieved with a SQUID is about 1 fT/Hz^0.5^ for a 1.5 cm^2^ loop with low-*T_c_* SQUID and about 30 fT/Hz^0.5^ with high-*T_c_* SQUID [82]. The final integrated system is incorporated in a portable Dewar.

## 5. Integrated GMR Systems for In Vivo/Vitro Biomagnetic Signal Detection

The brief overview on integrated GMR technologies in Section 3 demonstrates that it is a feasible strategy to employ integrated GMR sensors for ultra-low magnetic fields (nT/Hz^0.5^ to fT/Hz^0.5^) sensing. However, application of integrated GMR technology in vivo/vitro biomagnetic signal detection still faces great challenges, and has only been validated by a few research groups. Table 1 summarizes the reported studies successfully utilized integrated GMR system for in vivo/vitro biomagnetic signal sensing in recent years mainly by two research groups. The integrated GMR systems in Table 1 are mainly subject to the biomagnetic signal in the low-frequency band. There is no need for specific shielding in biomagnetic signal sensing with high intensity, such as action potential from the skeletal muscle [83]. But in detection of biomagnetic signal lower than pT level, such as MCG and MEG, shielding will be necessary to avoid the pollutions from the environmental noise (nT level). In the work by Pannetier-Lecoeur et al., the noise level of the integrated GMR and superconducting loop system at 77 K can reach fT, comparable to low-*T_c_* SQUID, and the final system is incorporated in a portable Dewar. All the SV sensors utilized in Table 1 work at room temperature with μm dimension. The reported successful applications of integrated GMR technology in biomagnetic signal detection are detailed in the following.

### 5.1. GMR-Superconducting Integrated Sensors for In Vivo MCG Measurements

**Figure 13:**
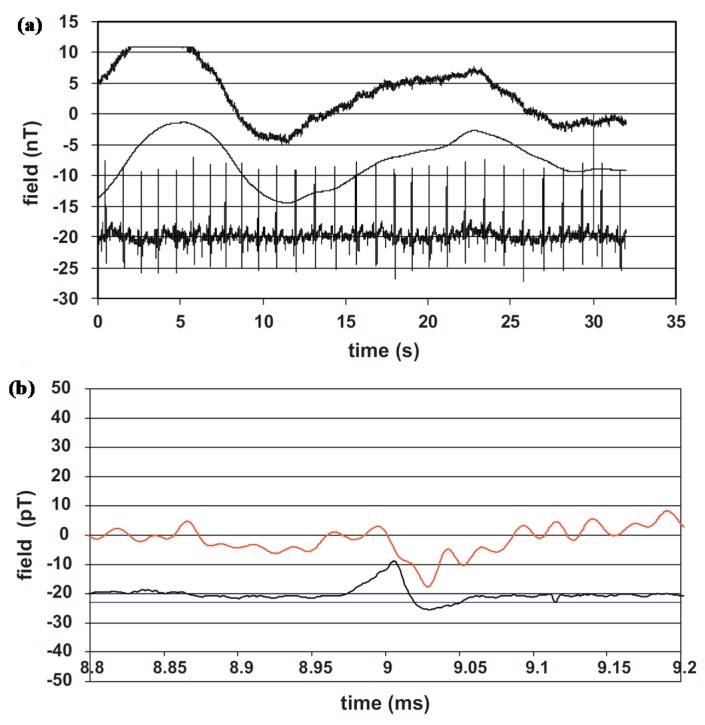
(**a**) MCG recorded on low-*T_C_* SQUID (upper curve) and mixed sensor (lower curve), simultaneously with the ECG; (**b**) Detailed MCG response (red) and the ECG peak as reference [27].

A GMR-superconducting integrated sensor (Figure 12) was developed by Pannetier et al. for the measurement of extremely low signals, such as MEG, and firstly reported in 2004 [26]. The stack of the GMR has the following composition [Si/SiO_2_/Ta(5)/Ni_81_Fe_19_(4)/Co_90_Fe_10_(1.2)/Cu(2.4)/Co_90_Fe_10_(2.4)/Ir_20_Mn_80_(10)/Ta(10)] (thicknesses are given in nm) and was patterned in a yoke-shape with a MR ratio of 4% at room temperature and of 9% at 4.2 K [26]. The small size prototype can be used at 77 K and is capable of measuring 30 fT/Hz^0.5^ or 1 fT/Hz^0.5^ at 4 K. Sensitivity of the sensor is represented by the local field enhancement due to the supercurrent flowing through the loop. The integrated system is incorporated in a portable Dewar to realize operation of the superconductor flux-to-field transformer at low temperature. This system suffers from thermal noise with a noise level of few fT/Hz^0.5^. Based on the integrated system, in vivo MCG measurements were presented (as shown in Figure 13) correlated with simultaneous recording of the electrocardiograph (ECG) later in shielded environment [27]. Although small peaks corresponding to the heart signals can be distinguished (Figure 13b), the noise level of the measurements remains too high.

**Figure 14:**
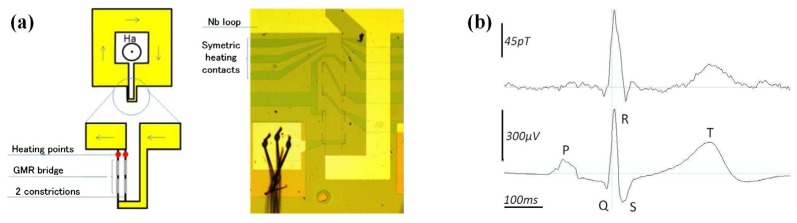
(**a**) Schematic of an antiparallel mixed sensor; (**b**) Simultaneous recordings of MCG (**top**) and ECG (**bottom**) averaged during 30 s with the mixed sensor reported in [84].

An improved structure design was proposed later to overcome the interference brought by a high noise level [83,84]. The technique is proposed on a modulation of the supercurrent passing through two constrictions in parallel of the loop by a local Joule heating. Figure 14a illustrates the parallel mixed-sensor design. MCG recordings were successfully recorded by this device. As presented in Figure 14b, the waveform feature fits well with simultaneous ECG recordings, where typical components (the T-wave and the QRS-complex) of the cardiac cycle were observed. This integrated sensor was fabricated either with a low-*T_C_* (niobium) loop, or with a high-*T_C_* (YBaCuO) loop. All the fabrication process can be realized by standard photolithography, ion beam etching and sputtering techniques. Although this integrated GMR/superconducting integrated sensor applied for BMSI still is at the proof-of-concept stage, the measurements of MCGs showed promising results for the integrated GMR sensor as a sensitive technique for BMSI applications. It is reasonable to expect that by reducing the distance from the sensor to the surface of the Dewar, detection of MEG responses can be realized.

### 5.2. Integrated GMR-Based Microprobes for Biomagnetic Response

The BMSI allow reference-free and coherent measurements, but the extremely low intensity requires the magnetic sensor to be placed as close as possible to the field sources. The merit that the GMR sensors can be fabricated at nanoscale size [21] makes it possible to realize measurements of biomagnetic signals within several tens of micrometers distance from the signal source. This will provide micron-size spatial resolution in neurophysiology studies. As summarized in Table 1, two research groups have reported successful validations of local recording of biomagnetic signals using integrated GMR-based silicon probes [21,22,23,28,85,86].

**Figure 15:**
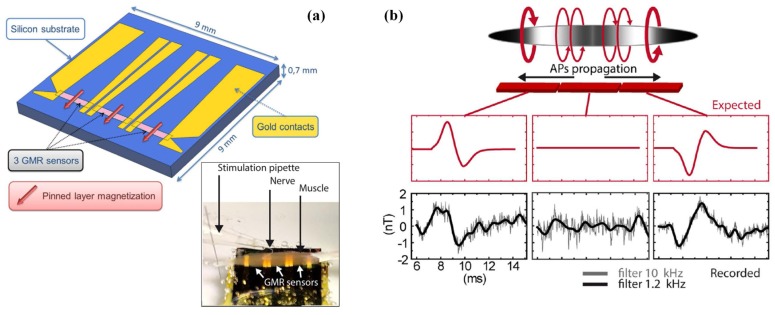
(**a**) Photography of the recording chamber and GMR sensor configuration; (**b**) Expected and recorded simultaneous magnetic signal on the three GMR-sensor derived from active potential after electrical stimulation of the nerve [85].

In the work by Barbieri et al. [85], a bio-compatible sensor integrated SV sensor was presented to record the magnetic fields derived from action potentials of a mouse skeletal muscle. As shown in Figure 15a, the proposed integrated GMR probe consists three aligned SV sensor of 1.7 mm × 400 μm on a silicon substrate, and a 1 cm-long soleus muscle was placed on the top of the magnetic probe. The recorded simultaneous magnetic fields by the three segments of the probe derived from active potential after electrical stimulation on the nerve are presented in Figure 15b. Magnetic field traces are averages over 500 trials and filtered with a low-pass filter. The tendency and the peak-to-peak amplitude (2.7 nT) of the detected magnetic fields agree well with the predictions. The results validate the suitability of integrated GMR-based probe for biomagnetic signal sensing, and confirm that the transmembrane currents contribute little to the net biomagnetic fields. This work provides essential tool to reveal the relationship between the local sources and the macroscopic MEG signals.

**Figure 16:**
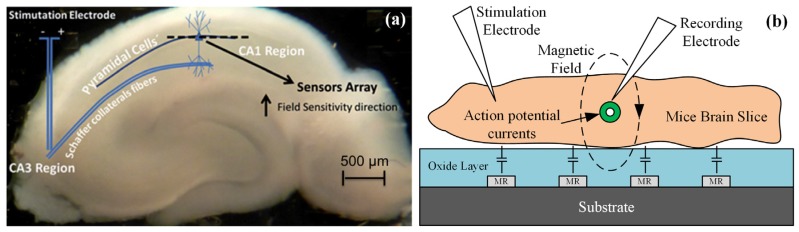
(**a**) Hippocampus slice with relative position of the sensor array with respect to the hippocampus structure [13]; (**b**) Schematic of the integrated GMR probe for neuronal measurement.

Another group focused on research in integrated GMR-based silicon probe applied for low order neuronal magnetic fields detection [13,21,22,23,28]. In their work, an integrated brain signal detector integrated with GMR sensors for the magnetic response of neurons from a mouse hippocampus brain slice has been proposed. The first generation of detector employs a planar array of 15 microfabricated MR sensing elements according to the arrangements of 3 × 50 μm^2^ (width × distance between electrical leads) [21,23]. The top pinned SV sensors are structured as followings: Si/Al_2_O_3_ 500/Ta 20/NiFe 25/CoFe 20/Cu 20/CoFe 22/MnIr 60/Ta 20/TiW(N_2_) 150 (thicknesses are given in Å) and the substrate is Si with Al_2_O_3_ layer. The microfabricated wafer is diced into individual magnetoresistive chip and wire-bonded to a ribbon flat cable with silicone gel for protection. The fabricated GMR sensor shows a sensitivity of 1.5%/mT. In the extracellular measurements, the planar GMR sensor array is placed under the excited brain slice (Figure 16), and the distance between them is about several micrometers. The magnetic field created in the SV sensor can be estimated at 6.5 nT.

**Figure 17:**
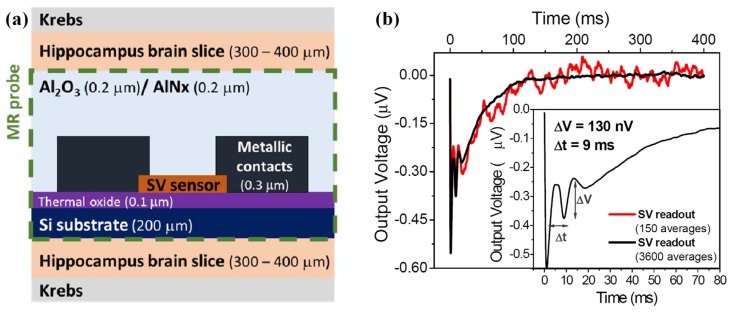
(**a**) Schematic view of the integrated GMR-based Si needle cross section; (**b**) Recording of SV sensor generated by the action potential currents resulting from the activation of multiple neurons in the CA1 region [28].

An improved detector combining Si needles and GMR sensors for neuronal magnetic field detection was developed based on the first generation to suit in vitro experiments on brain tissue [22,28]. As shown in Figure 2c, the GMR sensors in [28] are incorporated in the tip of micro-machined Si needles which could allow the detector to be inserted within the brain slice and get closer to the signal source with minimum damage to the brain slice. Figure 17a gives the schematic view of the needle. The bottom pinned SV sensors are structured as following: Si/SiO_2_ (100)/Ta (2.0)/ Ni_80_Fe_20_ (6.0)/ Ir_76_Mn_24_ (7.0)/ Co_80_Fe_20_ (3.3)/ Cu (2.5)/ Co_80_Fe_20_ (2.3)/ Ni_80_Fe_20_ (2.8)/ Ta (6.0) (thicknesses are given in nm and alloy compositions in at.%). 

In the in vitro experiments performed on mice brain slices, the SV sensor detects the magnetic field arising from the neuronal stimulation at a distance of 400 nm from the field source. As shown in Figure 17b, the recorded biomagnetic signal derived from stimulation is 33 nT based on the assumption of pure magnetic signal and the SV sensor sensitivity. The development of this integrated GMR platform will improve the electrophysiological researches. This work provides possible method to solve the problem that the intensity of biomagnetic field decreases greatly with increasing distance between the neuronal source and the detector.

**Figure 18:**
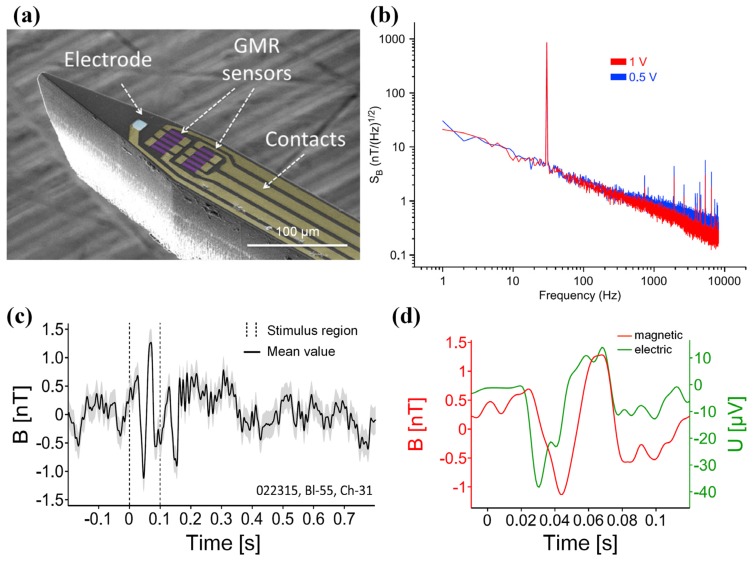
(**a**) Scanning electron microscopy picture of a magnetrode with GMR elements deposited on a silicon substrate; (**b**) Equivalent-field noise spectral density S_B_ from 1 Hz to 10 kHz of the proposed probe; (**c**) In Vivo neuronal magnetic signals on the magnetrode; (**d**) Magnification of the event-related-field around stimulus onset [86].

Recently, a joint research by the aforementioned two groups was carried out to record in vivo neuronal magnetic activity in the visual cortex of cats, and the results were reported in [86]. The developed needle-shaped magnetrode consists micron-sized (4 × 30 μm^2^), non-cooled GMR sensors and an electrode arranged in a meandering configuration on silicon substrate (200 μm thick) insulted by Si_3_N_4_/Al_2_O_3_ (Figure 18a). From the noise spectrum presented in Figure 18b we can see that, the probe sensitivity at a typical peak-to-peak input AC voltage of 500 mV achieves 7 nT/Hz^0.5^ (10 Hz). In the in vivo experiment, the magnetrode was directly inserted into the tissue to record the neuronal magnetic and electric response after stimulation (100 ms duration) simultaneously. The recorded neuronal magnetic field in Figure 18c shows that a magnetic response arises after stimulus onset, but a 20 ms delay exits compared with magnification signals in Figure 18d. The peak-to-peak amplitude of neuronal magnetic field achieves 2.5 nT. The results in Figure 18c,d validate the conduction delay between the retina and the related primary cortex. The work of recording inside magnetic fields provides possibility to better understand the extracranial MEG signals.

## 6. Conclusions

The inherent merits of GMR, which combines enhanced properties from multiple technologies, make integrated GMR systems good candidates for weak biomagnetic signal detection. Enormous interest has been focused on applications of integrated GMR in BMSI (ranging from pT/Hz^0.5^ to fT/Hz^0.5^ with the intensity decreasing with increasing distance). In this paper, the physical principles of GMR multilayers and electrophysiological origin of biomagnetic signals have been presented. Progress in GMR and challenges in point-of-use and point-of-care BMSI are highlighted, and the meeting point between them is presented accordingly. Strategies of integrated GMR technologies focused on 1/*f* noise reduction and sensitivity enhancement are discussed with relevant literature references, to provide guidance for future development of the integrated GMR technology in sub-pT biomagnetic signal detection. The state-of-art progress of integrated GMR technology for in vivo/vitro BMSI applications is reviewed as well.

As demonstrated in this review, with the increasing demands for portable BMSI devices in point-of-care and point-of-use applications, e.g., clinical diagnoses, HCI, education, and even entertainment, highly sensitive integrated GMR sensors are of enormous interest for high spatial and temporal biomagnetic signal detection, taking advantages of the high compatibility with standard CMOS process and size-independent sensitivity at room temperature, microminiature and low cost. With the various efforts focused on the integration of GMR and multiple technologies, its field sensitivity is being pushed to fT/Hz^0.5^. Although the integrated GMR technologies applied for BMSI are still at the proof-of-concept level and attempts were made to detect predictable and simple biomagnetic signals with nT to pT magnitude, the results show great potential for integrated GMR sensors as a sensitive technique for weak biomagnetic signal sensing. In addition to that, the local recording inside the tissues based on the integrated micro-sized GMR sensors offers an essential tool to further investigate and interpret external macroscopic biomagnetic signals, such as MEG.

Nevertheless, there are still challenges for GMR applied in BMSI. Further work may need to address the further integration with CMOS for real-time readouts, non-uniformity issues in the microfabrication process, size effect in the superconducting flux-transformer, and biocompatibility for quasi-static BMSI. The potential of integrated GMR technology in detection of low-frequency ultra-low biomagnetic signal seems to be far from being fully exploited.

## Figures and Tables

**Table 1:** Representative studies on integrated GMR system for biomagnetic signal detection.

References		Biomagnetic Signal	Sensors			Measured Distance	Noise Level & Sensitivity & Recorded Level
			Type	Size	Environment		
Pannetier-Lecoeur, M. et al.	[27,29]	In-vivo MCG of human body	GMR integrated a superconducting loop	SV sensor 2 μm width; superconducting loop 25 × 25 mm^2^	77 K & MSR	25~30 mm	3 fT/Hz^0.5^ & 3 pT/Hz^0.5^ & few pT
	[85]	In-vitro action potential of a mouse skeletal soleus muscle	3 SV sensors	1.7 mm long	Room temperature	NR	3.5 nT/Hz^0.5^ & 0.5 nT/Hz^0.5^ & PPA 2.7 nT
Joint research	[86]	In-vivo neuronal activity in the visual cortex of cats	SV sensor array	30 × 4 μm^2^		Inside the neuropil	NR & 7 nT/Hz^0.5^ at 10 Hz & PPA 2.5 nT
Freitas, P.P. et al.	[21,22]	In-vitro synaptic/action potential current in a mouse brain slice	15 SV sensors	30 × 2 μm^2^		1 μm	2~3 μV & 30 nT/Hz^0.5^ at 1 kHz & 2.5 μT
	[28]		2 SV sensors	40 × 2 μm^2^		1 μm	Few nV/Hz^0.5^ & 54 nT/Hz^0.5^ at 5 Hz & 33 nT

PPA is the abbreviation of Peak-to-peak amplitude.
